# Prescribing of diabetes medications to people with type 2 diabetes and chronic kidney disease: a national cross-sectional study

**DOI:** 10.1186/s12875-019-0915-x

**Published:** 2019-02-18

**Authors:** Jo-Anne Manski-Nankervis, Sharmala Thuraisingam, Janet K. Sluggett, Gary Kilov, John Furler, David O’Neal, Alicia Jenkins

**Affiliations:** 1 Department of General Practice, University of Melbourne, Carlton, Australia; 2 Centre for Medicine Use and Safety, Faculty of Pharmacy and Pharmaceutical Sciences, Monash University, Parkville, Australia; 3 Department of Medicine, St Vincent’s Hospital and University of Melbourne, Fitzroy, Australia; 4 Department of Endocrinology and Diabetes, St Vincent’s Hospital, Fitzroy, Australia; 5 NHMRC Clinical Trials Centre, University of Sydney, Camperdown, Australia

**Keywords:** Type 2 diabetes, Renal impairment, Chronic kidney disease, Prescribing, Guidelines, Anti-diabetes medications, Australia, General practice

## Abstract

**Background:**

Previous studies in general practice and hospital settings have identified that prescribing of non-insulin diabetes medications may be sub-optimal in people with type 2 diabetes (T2D) and renal impairment. Since these publications, a number of new medications have become available for the management of T2D. Study aims were to, in a cohort of Australians with T2D and renal impairment attending general practice, (1) investigate whether the prescribing of non-insulin diabetes medications is consistent with dosing adjustments recommended within current Australian Diabetes Society (ADS) guidelines; and (2) identify patient socio-demographic and clinical factors associated with at least one prescription of a non-insulin diabetes medication inconsistent with current ADS guidelines for medication doses.

**Methods:**

Cross-sectional study using data from the MedicineInsight general practice database managed by NPS MedicineWise. Patients with T2D who were aged 18 years and over, with an average eGFR< 60 ml/min/1.73m^2^ and at least one prescription of a non-insulin diabetes medication between 1st January 2015 and 30th June 2017 were included. Descriptive statistics were used to summarise patient characteristics and medication use. Marginal logistic regression models were used to estimate associations between sociodemographic and clinical factors and prescribing of ≥1non-insulin diabetes medicine not consistent with ADS guidelines.

**Results:**

The majority of the 3505 patients included (90.4%) had an average eGFR of 30-59 ml/min/1.73m^2^. In terms of absolute numbers, metformin was the medication most frequently prescribed at a dose not consistent with current ADS guidelines for dosing in renal impairment (*n* = 1601 patients), followed by DPP4 inhibitors (*n* = 611) and sulphonylureas (*n* = 278). The drug classes with the highest proportion of prescriptions with dosage not consistent with ADS guidelines were SGLT2 inhibitors (83%), followed by biguanides (58%) and DPP4 inhibitors (46%). Higher HbA1c, longer known diabetes duration and diagnosis of retinopathy were associated with receiving ≥1prescription with a dosage not consistent with guidelines.

**Conclusions:**

Prescribing of non-insulin diabetes medications at doses inconsistent with current ADS guideline recommendations for dosing adjustments for people with renal impairment was common. Further research is needed to understand how general practitioners access, interpret and apply the ADS guidelines and the impact this may have on patient outcomes.

**Electronic supplementary material:**

The online version of this article (10.1186/s12875-019-0915-x) contains supplementary material, which is available to authorized users.

## Background

The burden of type 2 diabetes (T2D) is increasing worldwide. The International Diabetes Federation has recently reported that 425 million, or 9%, of the world’s population aged 20 to 79 years has diabetes and this is expected to increase to 629 million by 2045 [1]. In high income countries approximately 90% of people with diabetes will have T2D [1].

In Australia, more than one million people have been diagnosed with T2D and 270,000 are estimated to have co-existing chronic kidney disease (CKD) [2, 3]. The majority of Australians with T2D receive their medical care in general practice, with more than five million encounters between general practitioners (GPs) and people with diabetes taking place annually [4]. Australian guidelines for the management of T2D in general practice recommend pharmacological management to optimise blood glucose levels to reduce the risk of complications if lifestyle measures have not achieved the recommended glycaemic targets [5]. The Australian Diabetes Society (ADS) has provided specific guidance on the dosing of anti-hyperglycaemic medications in people with renal impairment [6], warning that pharmacokinetic changes may occur resulting in increased risk of hypoglycaemia and other side-effects. These risks are likely to be most evident for people with CKD Stages 4 and 5 (estimated glomerular filtration rates (eGFR) < 30 and < 15 ml/min/1.73m^2^ respectively).

Several general practice and hospital studies have raised concerns about prescribing of glucose control-related diabetes medications to people with renal impairment which are inconsistent with guideline recommendations and product information [7–9] and which may place people at risk of hypoglycaemia and adverse drug events (ADEs). Limitations of these studies are that they have relied on self-report rather than routinely collected medication data from administrative datasets and several have not considered the prescribed dose in the analyses. Since the publication of these papers, there have been changes to the guidance on use of these medications in renal impairment [6], new classes of medication are available [10], as well as additional research which indicates that some of the medications not recommended for use in renal impairment may in fact have benefits for reducing progression of CKD, cardiovascular disease (CVD) and mortality [11–15].

Our previous work exploring prescribing of diabetes medications in people with T2D and renal impairment found that 48.3% of the cohort of 9624 people were prescribed at least one non-insulin diabetes medication at a dose inconsistent with ADS guidelines in October 2014 to September 2015 [16]. Since this time, the indications for SGLT2 inhibitors in the Pharmaceutical Benefits Scheme (PBS; Australian government scheme to subsidise the costs of medications) have been extended and more fixed-dose combination products have become available. We were interested to explore whether these factors, as well as whether using an average of 2 eGFR readings rather than a single reading to define appropriate prescribing, using diabetes-specific co-morbidities as co-variates and being more conservative utilising minimum dosage when a range of dosages was recorded in the prescription, would have resulted in a difference in the proportion of people prescribed a medication inconsistent with ADS guidelines.

The aims of this study were to, in a cohort of Australians with T2D and renal impairment attending general practice, (1) investigate whether the prescribing of non-insulin diabetes medications is consistent with dosing adjustments recommended within current Australian Diabetes Society (ADS) guidelines; and (2) identify patient socio-demographic and clinical factors associated with at least one prescription of a non-insulin diabetes medication inconsistent with current ADS guidelines for medication doses.

## Methods

### Study sample

This study was conducted utilising data from MedicineInsight. This national database managed by NPS MedicineWise was established to support quality improvement in Australian general practice and post-market surveillance of medicines and has now been made accessible for approved research. MedicineInsight collects demographic, anthropometric, pathology requests and results, radiology test requests, and medical history data, from GP clinical information systems (CIS), including Medical Director and Best Practice. The data are only re-identifiable in the clinic. Data utilised for the present study were collected from 557 Australian general practices, located in every Australian state and territory, and are including more than 3.8 million patient records [17, 18].

**Fig. 1:**
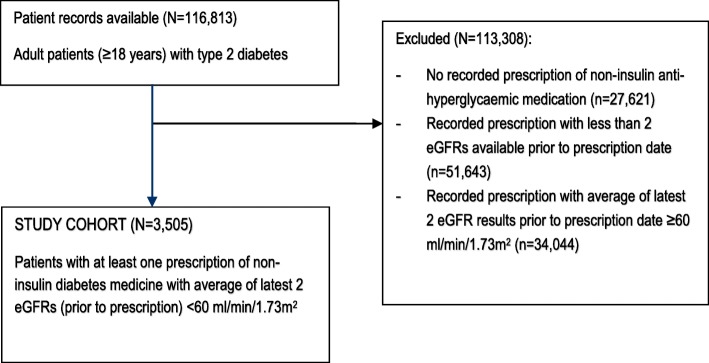
Patient cohort

The study population consisted of patients with a recorded diagnosis of T2D who were aged 18 years and over. The final study population was identified using a step-wise approach (Fig. 1). Patients were excluded if they had no recorded prescription of a non-insulin diabetes medicine between 1st of January 2015 and 30th of June 2017. Patients with a recorded prescription who did not have at least two eGFR measurements prior were also excluded. The final cohort consisted of patients with at least one recorded prescription with an average eGFR < 60 ml/min/1.73m^**2**^ prior to prescription. If more than one prescription was issued for the same medicine, the most recent prescription was included in the analysis. The average eGFR was calculated from the latest two eGFRs taken prior to the prescription being issued. eGFRs from the 1st of January 2014 to 30th June 2017 were included in this analysis (mean 168 days between eGFR measurements). eGFR results were identified using LOIN-C codes [19, 20] and text searches on test name.

### Medication use

The prevalence of non-insulin diabetes medications (identified by ATC code A10B) and prescribed doses were determined by identifying the most recent prescription during the study period (between 1st of January 2015 and 30th of June 2017). Conformance of prescriptions to current ADS guideline recommendations for medication dosage in patients with T2D with renal impairment was determined [6]. Prescribing recommendations for empagliflozin were taken from product information lodged with the Therapeutic Goods Administration (TGA) [21] as this medication was not available in Australia at the time the ADS guidelines were issued. The ADS guidelines provide maximum recommended doses of medications by CKD stage. A prescription was labelled as not consistent with guidelines if it was prescribed when ADS guidelines stated that it was not recommended or should be avoided, or when dosed in excess of that recommended [6]. Where medication doses were specified as a range on the prescription, the minimum of the range was used in the analysis to be conservative. Prescriptions with no dosage recorded, or dosage documented as “mdu” or “immediate”, were coded as missing and excluded from the analysis focusing on associations of prescribing to guidelines. The components of combination products were considered as separate medications. A full list of non-insulin diabetes medications available in Australia is provided in the Additional file 1: Table S1.

### Clinical characteristics

The covariates in this study included gender, rurality (as per the postcode of the patient’s residence using the Australian Bureau of Statistics Australian Statistical Geography Standard [22]), self-reported indigenous status, diabetes duration, insulin use, HbA1c and diabetes specific comorbidities such as hypertension, coronary heart disease (CHD), heart failure (HF), stroke, amputation, impaired vision and retinopathy. Conditions were included if they were ever recorded in the CIS as a diagnosis or reason for encounter or reason for prescription. Hypertension was also coded if the most recently recorded systolic blood pressure was greater than 140 mmHg or diastolic blood pressure greater than 90 mmHg. The HbA1c utilised was the most recently recorded. Diabetes duration (years) was defined as time from recorded onset to end of study period.

### Statistical analyses

Descriptive statistics were used to summarise the characteristics of patients included in the study. Medication use was quantified by drug class, active ingredient and type of combination therapy. The count and proportion of patients in the cohort prescribed metformin, sulphonylureas, DPP4 inhibitors, thiazolidinediones, incretin mimetics, SGLT2 inhibitors, acarbose and combination therapies were summarised. The proportion of these prescriptions not consistent with ADS guidelines [6] were stratified by eGFR category (45–59, 30–44 and < 30 ml/min/1.73m^2^). Marginal logistic regression models were used to estimate the odds of a patient being prescribed at least one non-insulin diabetes medication at a dose not consistent with ADS guidelines for the following socio-demographic and clinical factors: gender, rurality, indigenous status, diabetes duration, insulin use, HbA1c and diabetes-related complications (hypertension, coronary heart disease, heart failure, stroke, amputation, impaired vision and retinopathy). Age was originally included in the model but was removed due to multicollinearity with HbA1c and diabetes duration. The model with age removed resulted in better fit of the data with variance inflation factors reduced to within acceptable limits and improved stability. Both unadjusted and adjusted odds ratios were determined with 95% confidence intervals and *p*-values. Robust standard errors were used to account for the correlation in prescribing patterns within practices. Interaction terms were added to the regression model to determine whether associations between patient factors and at least one prescription not consistent with ADS guidelines varied by the three most frequently prescribed medication classes (metformin, sulphonylureas and DPP4 inhibitors).

A number of alternate guidelines and product information statements advise prescribing decisions for people with renal impairment should be based on creatinine clearance, not eGFR. In a sensitivity analysis, creatinine clearance was estimated using the Cockcroft-Gault equation^1^ [23], and the average of the latest two of these results prior to the prescription was calculated and the analyses repeated utilising this instead of eGFR.^2^ Prescriptions with missing dosages and frequency were coded as missing data. All data management and analyses were conducted using SAS Enterprise Guide 7.1 (Cary, NC USA, 2015) and STATA (StataCorp) version 13.1.

## Results

### Patient characteristics

Details of 116,813 people with T2D were available from the MedicineInsight database during the study period. Of these, 3505 (3.9%) were included in this study (Fig. 1).

The mean age of the cohort was 77.4 years and consisted of more males than females (52% vs. 48%) (Table 1). Close to 60% of patients were from major cities and, of the 3054 (87.1%) with a BMI recorded, over 50% were classified as obese based on their BMI (> 30 kg/m^2^). More than half (54%) had an average eGFR between 45 and 59 ml/min/1.73 m2 and just over a third had an average eGFR between 30 and 44 ml/min/1.73m^2^. The average duration of diabetes was 12 years and average HbA1c 7.3% (56 mmol/mol). Over 80% of patients had a recorded diagnosis of hypertension. Comparison of characteristics of patients who were and were not included in the study showed patients excluded from the study were younger, with a shorter duration of diabetes and fewer complications (Table 1). Of these, one in five patients (22% of those with a recorded eGFR) showed some evidence of renal impairment but the timing or limited number of eGFR tests meant that they were not eligible for inclusion in this study.

**Table 1:** Sociodemographic characteristics of patients included in, and excluded from, the study

Sociodemographic characteristic^a^	Included in the study *N* = 3505	Excluded from study *N* = 113,308
	n (%)	n (%)
Gender		
Male	1838 (52.4)	60,572 (53.5)
Female	1667 (47.6)	52,709 (46.5)
Not known		27 (0.02)
Age (years)		
Age, mean (SD)	77.4 (9.0)	66.8 (13.5)
Age, median (Q1, Q3)	78 (72, 84)	68 (58, 77)
Age group (years)		
18–29	–	892 (0.8)
30–39	7 (0.2)	3113 (2.8)
40–49	17 (0.5)	8550 (7.6)
50–59	99 (2.8)	18,883 (16.7)
60–69	521 (14.9)	30,687 (27.1)
70–79	1301 (37.1)	30,750 (27.1)
≥ 80	1560 (44.5)	20,433 (18.0)
Indigenous status		
Aboriginal and/or Torres Strait Islander	66 (1.9)	3033 (2.7)
Not Aboriginal nor Torres Strait Islander	2734 (78.0)	87,427 (77.2)
Not known	705 (20.1)	22,848 (20.2)
Rurality^b^		
Major cities of Australia	2039 (58.6)	65,244 (57.6)
Inner Regional Australia	975 (28.0)	32,422 (28.6)
Outer Regional Australia	413 (11.9)	13,536 (12.0)
Remote Australia	41 (1.2)	1207 (1.1)
Very Remote Australia	14 (0.4)	294 (0.3)
Not known	23 (0.7)	605 (0.5)
Duration of known diabetes		
Duration of diabetes (years), mean (SD)	12.2 (7.9)	8.7 (6.8)
Duration of diabetes (years), median (Q1, Q3)	11.2 (6.0, 16.8)	7.1 (3.5, 12.4)
Not known	740 (21.1)	27,357 (24.1)
HbA1c^c^, mean (SD)		
% (NGSP^d^)	7.3 (1.3)	7.1 (1.4)
mmol/mol (IFCC^e^)	56.1 (14.2)	53.7 (15.3)
Not known	750 (21.4)	37,121 (32.8)
Average Glomerular filtration rate (ml/min/1.73m^2^)		
≥ 60	0	80,127 (78.0)
45–59	1909 (54.5)	12,533 (12.2)
30–44	1261 (36.0)	6985 (6.8)
15–29	290 (8.3)	2465 (2.4)
< 15	45 (1.3)	616 (0.6)
Not known		10,582 (9.3)
BMI (kg/m^2^)		
Underweight (< 18.5)	11 (0.4)	465 (0.5)
Normal (18.5 to < 25)	386 (12.6)	12,240 (12.3)
Overweight (25 to 30)	1023 (33.5)	30,069 (30.3)
Obese (> 30)	1634 (53.5)	56,392 (56.9)
Not known	451 (12.9)	14,142 (12.5)
Comorbidities		
Hypertension	2855 (81.5)	75,500 (66.6)
Coronary Heart Disease (CHD)	982 (28.0)	19,483 (17.2)
Heart failure (HF)	440 (12.6)	6250 (5.5)
Stroke	394 (11.2)	7300 (6.4)
Amputation	29 (0.8)	632 (0.6)
Impaired vision	129 (3.7)	2565 (2.3)
Retinopathy	182 (5.2)	3411 (3.0)
Medications		
On insulin^f^	949 (27.1)	4626 (29.8)
On erythropoietin agonist^g^	12 (0.3)	113 (0.1)

Abbreviations: *Q1* 25th percentile, *Q3* 75th percentile

^a^Note: percentages may not sum to 100 due to rounding

^b^Rurality was assigned according to the postcode of the patient’s residence using the Australian Bureau of Statistics Australian Statistical Geography Standard

^c^Previously referred to as the National Glycohemoglobin Standardization Program

^d^International Federation of Clinical Chemistry and Laboratory Medicine

^e^Note: average of latest 2 eGFR results prior to prescription

^f^This includes patients who had a valid insulin prescription at 30/06/17, patients who had an insulin prescription in the past and those with an insulin prescription with missing dosages

^g^Erythropoietin agonists are erythropoietin, darbepoetin alfa and methoxy polyethylene glycol-epoetin beta (ATC codes B03AX01–3). This includes patients with a valid prescription at 30/6/17, patients who had a prescription in the past and those with a prescription with missing dosage

### Prescription of non-insulin diabetes medications by drug class for patients with average eGFR < 60 ml/min/1.73m^2^

The prescription of non-insulin diabetes medications by drug class is summarised in Table 2. Biguanide (metformin) was the most commonly prescribed medication class (81%), followed by sulphonylureas (52%) and DPP4 inhibitors (39%). Just over a third of patients (38%) were prescribed two non-insulin glucose-lowering diabetes medications and 16% were prescribed three. Of the combination therapies (e.g. medication from two or more classes combined in one dosage form), biguanide in combination with DPP4 inhibitors were most commonly prescribed (16% of all patients).

**Table 2:** Prescription of non-insulin diabetes medication by drug class for patients with T2D and average eGFR < 60mlk/min/1.73m^2^

Medication Class	n (%) patients prescribed medication	n (%) of patients prescribed medication that is not consistent with dosing recommendations in ADS dosing guidelines			
		eGFR < 60 ml/min/1.73m^2^ *n* = 3505 patients	eGFR 45–59 ml/min/1.73m^2^ *n* = 1909 patients	eGFR 30–44 ml/min/1.73m^2^ *n* = 1261 patients	eGFR < 30 ml/min/1.73m^2^ *n* = 335 patients
Biguanide (metformin)	2853 (81.4)	1601 (58.1)	702 (25.5)	762 (27.7)	137 (5.0)
Sulphonylureas	1818 (51.9)	278 (16.0)	112 (6.5)	66 (3.8)	100 (5.8)
DPP4 inhibitors^a^	1371 (39.1)	611 (46.4)	374 (28.4)	174 (13.2)	63 (4.8)
Thiazolidinediones (TZDs)	106 (3.0)	< 5	0	0	< 5
Incretin mimetics	150 (4.3)	12 (13.6)	< 5	< 5	6 (6.8)
SGLT2 inhibitors^b^	218 (6.2)	180 (82.6)	125 (57.3)	51 (23.4)	< 5
Acarbose	24 (0.7)	< 5	< 5	0	< 5
Combination products (2 or more medications in a single formulation)^c^					
Biguanide & Sulfonylureas	34 (1.0)	31 (93.9)	23 (69.7)	8 (24.2)	< 5
Biguanide & DPP4 inhibitors	574 (16.4)	475 (84.1)	286 (50.6)	156 (27.6)	33 (5.9)
Biguanide & TZDs	< 5	< 5	< 5	< 5	0
Biguanide & SGLT2 inhibitors	54 (1.5)	47 (94)	36 (72.0)	11 (22.0)	0

^a^ Dipeptidyl peptidase 4 inhibitors

^b^ Sodium glucose co-transporter 2 inhibitors

^c^Medications included in the combination products are also included in the single medication classes above

Note: cell counts less than 5 suppressed

In terms of absolute numbers, metformin was the medication most frequently prescribed at a dose not consistent with current ADS guidelines for dosing in renal impairment (*n* = 1601 patients), followed by DPP4 inhibitors (*n* = 611) and sulphonylureas (*n* = 278). The drug classes with the highest proportion of prescriptions with dosage not consistent with ADS guidelines were SGLT2 inhibitors (83%), followed by biguanides (58%) and DPP4 inhibitors (46%). Approximately four in five patients (79%) prescribed a combination product had a dose prescribed that was not consistent with guidelines. The drug combination with the highest proportion of patients prescribed medication or doses inconsistent with guidelines was biguanide and sulfonylureas (94%), followed by biguanide and SGLT2 inhibitors (90%) and biguanide and DPP4 inhibitors (84%). Data for individual medications within each drug class is provided in Additional file 2: Table S2.

The proportion of patients prescribed at least one medication with a dosage inconsistent with the ADS guidelines for people with renal impairment was 59% (2057/3505). If all metformin prescriptions were excluded, the proportion of people prescribed at least one medicine with a dosage inconsistent with ADS guidelines reduced to 39% (962/2498 patients). There were 231 (7% of total cohort) patients with eGFR < 30 ml/min/1.73m^2^ with at least one prescription at a dose not consistent with guidelines.

### Patient factors associated with at least one prescription of a non-insulin diabetes medication at a dose inconsistent with guidelines

Table 3 shows associations between patient demographic and clinical factors and the likelihood of at least one non-insulin diabetes medication prescription at a dose inconsistent with guidelines. There is evidence of an association between increasing diabetes duration and being prescribed at least one non-insulin diabetes medication with a dose inconsistent with ADS guidelines. For every one-year increase in diabetes duration, the odds of at a patient prescribed at least one prescription at a dose inconsistent with guidelines increased by 2% (OR 1.02, 95% CI 1.01 to 1.04). Similarly, for every 1% (11 mmol/mol) increase in HbA1c, the odds of at least one prescription inconsistent with guidelines increased by 28% (OR 1.28, 95% CI 1.15 to 1.41).

**Table 3:** Odds ratio for factors associated with ≥1 prescription of non-insulin diabetes medication inconsistent with guidelines

	N	At least one prescription of a non-insulin diabetes medication inconsistent with dosing recommendations n (%)	Unadjusted			Adjusted		
			OR^€^	95% CI	*P* value	OR^€^	95% CI	P value
Gender								
Males	1792	1090 (60.8)	1.20	(1.05 to 1.37)	0.008	1.14	(0.92 to 1.40)	0.22
Females (reference group)	1624	915 (56.3)	1			1		
Rurality					0.81			0.42
Major cities of Australia (reference group)	1977	1150 (58.2)	1			1		
Inner regional Australia	955	568 (59.5)	1.05	(0. 88 to 1.24)		1.02	(0.81 to 1.29)	
Outer regional Australia	406	237 (58.4)	0.99	(0.80 to 1.24)		1.01	(0.73 to 1.38)	
Remote and very remote Australia	55	35 (63.6)	1.26	(0.73 to 2.17)		1.57	(0.77 to 3.24)	
Indigenous status								
Aboriginal and/or Torres Strait Islander	62	36 (58.1)	0.94	(0.56 to 1.58)	0.83	0.88	(0.44 to 1.74)	0.84
Not Aboriginal nor Torres Strait Islander	2679	1580 (59.0)	1			1		
Diabetes duration, (years)	2765		1.03	(1.02 to 1.04)	< 0.001	1.02	(1.01 to 1.04)	0.04
Insulin use								
Yes	922	593 (64.3)	1.37	(1.19 to 1.59)	< 0.001	1.07	(0.85 to 1.35)	0.58
No	2494	1412 (56.6)	1			1		
HbA1c %	2755		1.22	(1.14 to 1.31)	< 0.001	1.28	(1.15 to 1.41)	< 0.001
Comorbidities								
Hypertension	2794	1644 (58.8)	1.03	(0.86 to 1.24)	0.72	1.09	(0.82 to 1.45)	0.54
No hypertension	622	361 (58.0)	1			1		
CHD	955	566 (59.3)	1.04	(0.91 to 1.19)	0.59	1.00	(0.81 to 1.24)	0.97
No CHD	2461	1439 (58.5)	1			1		
HF	429	235 (54.8)	0.83	(0.67 to 1.02)	0.08	0.80	(0.59 to 1.09)	0.16
No HF	2987	1770 (59.3)	1			1		
Stroke	384	209 (54.4)	0.82	(0.67 to 1.01)	0.06	0.81	(0.59 to 1.11)	0.18
No stroke	3032	1796 (59.2)	1			1		
Amputation	28	17 (60.7)	1.07	(0.50 to 2.30)	0.86	1.54	(0.39 to 6.10)	0.54
No amputation	3388	1988 (58.7)	1			1		
Impaired vision	128	69 (53.9)	0.83	(0.58 to 1.19)	0.31	0.91	(0.57 to 1.44)	0.68
No impaired vision	3288	1936 (58.9)	1			1		
Retinopathy	180	131 (72.8)	1.94	(1.38 to 2.72)	< 0.001	1.91	(1.18 to 3.09)	0.009
No retinopathy	3236	2303 (71.2)	1			1		

€ Odds ratio estimated using univariable and multivariable logistic regression with robust standard errors

Note: percentages may not sum to 100 due to rounding

Abbreviations: *CHD* coronary heart disease, *HF* heart failure

### Associations between patient factors and at least one diabetes medication prescription inconsistent with guidelines by drug class

The association between higher HbA1c and receiving at least one prescription with a dosage inconsistent with guidelines remains when the analysis is stratified by biguanides and sulphonylurea drug classes.^3^ The magnitude of the association between HbA1c and at least one prescription with dose inconsistent with guidelines is slightly larger for those prescribed biguanides (OR 1.48, 95% CI 1.29 to 1.87) compared to those prescribed sulphonylureas (OR 1.24, 95% CI 1.08 to 1.42). The association between known diabetes duration and receiving at least one prescription with dosing inconsistent with guidelines was only significant for those prescribed metformin (OR 1.03, 95%CI 1.01 to 1.05). There was a lack of evidence of an association between any of the clinical and patient sociodemographic factors and at least one prescription with dosing not consistent with ADS guidelines for those on DPP4 inhibitors.

### Sensitivity analyses

Results remained unchanged when the average creatinine clearance, calculated using the Cockcroft-Gault equation, was used to determine the proportion of prescriptions with a dosage that were not consistent with ADS guidelines (results available on request).

## Discussion

We explored prescription of non-insulin diabetes medications in primary care for a large sample of people with T2D and renal impairment. The majority of people included in the study were aged over 70 years and had an eGFR consistent with Stage 3 CKD, as opposed to Stage 4 or 5 CKD where the risk of harm associated with a prescription not consistent with guidelines are likely to be greatest. Overall, 59% of the cohort were found to have been prescribed at least one non-insulin diabetes medication that was not consistent with 2014 ADS guidelines for prescribing among people with diabetes and renal impairment, which is higher than that found in a previous study when less stringent criteria for renal impairment were utilised [16]. The drug classes with the highest number of patients categorised as having been issued a prescription not consistent with ADS guidelines were metformin, followed by DPP4 inhibitors and sulphonylureas. These prescriptions were not associated with gender, rurality, indigenous status or comorbidities, but were more likely to be prescribed to those with longer diabetes duration, higher HbA1c and a diagnosis of retinopathy.

Caution should be applied when interpreting these results in context of appropriateness of prescribing. Guidelines are recommendations based on evidence and consensus opinion and are based on appropriateness for a population. There are risks associated with measuring adherence to guidelines. To quote Norma and Eva [24]: “…adherence to prescribed practices of care may be, in some sense, optimal at a population level, but at an individual level, experienced physicians may deliberately and systematically depart from these guidelines to recognise individual patient needs.” This may be the case for the medications reviewed in this study.

Metformin was the most frequently prescribed medication to people with T2D and renal impairment in this study. Guidelines currently recommend dose reduction and cessation for this population because of a concern about lactic acidosis [6]. These vary in terms of the eGFR threshold for dose reduction, although there is consensus that metformin not be used in people with eGFR < 30 ml/min/1.73m^2^ [6, 25–27]. However, systematic reviews have concluded that lactate levels and risk of lactic acidosis do not differ appreciably in patients with CKD taking metformin compared to other oral diabetes medications [28] and there are no data from prospective comparative trials or observational cohort studies that support the hypothesis that metformin is associated with an increased risk of lactic acidosis [29]. In terms of potential benefits, a recent systematic review has shown that, in people with T2D and eGFR 30-59 ml/min/1.73m^2^, being prescribed metformin is associated with reduced all-cause mortality as well as reduced hospitalisation re-admission for heart failure [12]. In addition, SGLT2 inhibitors had the highest proportion of patients with prescriptions that were deemed inconsistent with guidelines and are currently recommended to be avoided in people with an eGFR < 45 ml/min/1.73m^2^. However, they may have benefits in renal impairment, independent of glycaemia, in terms of reducing cardiovascular events, albuminuria and decline of eGFR [13–15, 30].

We also found that the mean HbA1c was 7.3%. Relatively tight HbA1c control in an older population may be of concern particularly for patients prescribed sulphonylureas and insulin as these medications are associated with increased risk of hypoglycaemia, which is associated with increased risk of cardiovascular events and death [31, 32]. Individualisation of HbA1c targets is recommended by national [33] and international bodies [34, 35].

Nearly four in five patients (79%) prescribed a fixed-dose combination product had a dose prescribed that was not consistent with guidelines. Whilst it is convenient and cost-effective for patients to take a combination product rather than multiple separate products, our findings indicate that increased attention to individual doses within these products may be indicated. The Australian Medicines Handbook currently recommends that combination products should not be utilised until patients have been stabilised on similar doses of the included drugs [10], however research suggests that this is often not the case in older people with T2D [36]. This may be worth further investigation in this cohort.

Finally, it is important to acknowledge that the safety implications of nonconsistency with guidelines vary according to drug class and degree of the severity of renal impairment. For example, with metformin there is an increased risk of lactic acidosis with an eGFR of less than 30 ml/min/1.73m^2^, as opposed to SGLT2 inhibitors where the issue is not so much one of safety but that these agents are not effective in lowering glucose levels at a reduced eGFR. Approved linkage of the MedicineInsight data to hospital and mortality datasets to explore whether prescribing of diabetes medications that are not consistent with guidelines are associated with adverse patient outcomes is warranted.

### Strengths and limitations

The strengths of this study include the utilisation of a large, national ‘real world’ general practice dataset from 557 Australian general practices. We only included patients with an average eGFR based on two readings of < 60 m/min/1.73m^2^ prior to prescription so that patients included would be more likely to have a diagnosis of CKD according to Kidney Health Australia guidelines. This reduced the likelihood of aberrant eGFR results due to acute conditions such as dehydration secondary to infection being classified as CKD. We also characterised whether prescriptions were consistent with guidelines utilising eGFR results which would have been available to the GP preceding the issuing of either a new prescription or a repeat prescription.

There are several limitations which are important to acknowledge. It is important to acknowledge that we did not assess overall quality of consistency with treatment guidelines for all people with T2D and CKD, as we only focused on dosing of non-insulin glucose lowering diabetes medications. Secondly, limitations may exist with regards to the quality of data stored in an extractable format in the general practice electronic medical records. For example, medications that were marked as non-current were excluded from this study, but there may have been medications that had been ceased but not marked as non-current in the medical record. This may have resulted in a higher proportion of prescribing not consistent with guidelines. We had access to eGFR results and comorbidities recorded in extractable fields within the practices’ CIS, but additional information recorded elsewhere in the medical record, for example within scanned letters or discharge summaries, or if not provided by pathology providers in an extractable format, were not able to be assessed. Furthermore, only 4% of people with T2D prescribed at least one non-insulin diabetes medication were found to have two eGFR results < 60 ml/min/1.73 m2 prior to prescription. This is a low proportion given the estimated prevalence of CKD in this population and guideline recommendations that eGFR is measured at least once a year in people with T2D and CKD [33]. Possible explanations for this finding include: the results provided by the laboratory were in an incompatible format for electronic upload into the CIS, the patient did not have two eGFR tests performed during the study period and prior to the most recent prescription of a non-diabetes medication, or eGFR tests were performed by other health professionals and as a result were not recorded in an extractable field in the GP CIS. Finally, we calculated CrCl using actual body weight not lean body weight and this means we may have overestimated CrCl and renal function amongst people who were obese.

The MedicineInsight dataset consists of unique patient records rather than individuals and as a result there is a small possibility that patients attending multiple practices may have been counted more than once. It is also possible that a small percentage of patients who did not have T2D were prescribed metformin to control obesity or insulin resistance and may have been included in this study. There were also a number of lean people in this dataset (13% of people had a BMI in the underweight or normal category) and it is possible that some may have been misclassified as having T2D, and in fact have another condition such as latent autoimmune diabetes of adults (LADA) or type 1 diabetes. Some people with type 1 diabetes may also be prescribed metformin, particularly if overweight or obese or insulin resistant [37], and may have been included in this dataset if their records indicated an erroneous diagnosis of type 2 diabetes. In 2016–17, the MedicineInsight dataset included 5.9% of general practices and 7.5% of GPs in Australia [38]. The general practices contributing data to MedicineInsight do so on a voluntary basis and are participating in a quality improvement program and as such may not be representative of Australian general practice.

## Conclusion

In 3505 people with T2D and renal impairment attending an Australian general practice in the MedicineInsight dataset, a high percentage (59%) were found to have been prescribed at least one non-insulin diabetes medication that was not consistent with 2014 ADS guidelines. Further research is required to understand how general practitioners access, interpret and apply the ADS guidelines, the reasons for prescribing that is not consistent with guidelines and the impact this may have on patient outcomes. Approved linkage of MedicineInsight data to hospital and mortality datasets to explore whether prescribing of diabetes medications that are not consistent with guidelines are associated with adverse patient outcomes is warranted, given evolving research which demonstrates that prescribing outside of these guidelines may have benefits in terms of progression of CKD, CVD and mortality. This knowledge could be utilised to inform quality improvement strategies to optimise prescribing, and could be targeted to people with longer duration of diabetes and higher HbA1c who are more likely to be prescribed a non-insulin diabetes medication at a dose inconsistent with current ADS guidelines.

## Additional files

Additional file 1: **Table S1.** List of non-insulin diabetes medications available in Australia. This table summarises non-insulin diabetes medications (generic and brand names) available in Australia at the time this study was conducted. (DOCX 16 kb)

Additional file 2: **Table S2.** Prescription of non-insulin diabetes medications by eGFR for patients with average eGFR <60 ml/min/1.73m^2^. This table summarises the prescription of non-insulin diabetes medications by average eGFR consistent with stage 3a, 3b, 4 and 5 chronic kidney disease. (DOCX 24 kb)

## Abbreviations

ADE: Adverse drug event
ADS: Australian Diabetes Society
BMI: Body mass index
BMI: Body mass index
CHD: Coronary heart disease (CHD)
CIS: Clinical Information System
CKD: Chronic kidney disease
CrCl: Creatinine clearance
CVD: Cardiovascular disease
DPP4 inhibitor: Dipeptidyl peptidase-4 inhibitor
eGFR: Estimated glomerular filtration rate
GP: General practitioner
HbA1c: Glycated haemoglobin
HF: Heart failure
LADA: Latent autoimmune diabetes of adults
NPS: National Prescribing Service
SGLT2 inhibitor: Sodium glucose co-transporter 2 inhibitor
T2D: Type 2 diabetes
TGA: Therapeutic Goods Administration

## Footnotes

1 Cockcroft Gault formula: Creatinine Clearance (CrCl) (ml/min) = {(140 - Age) x wt (kg) x F} / (Plasma Creatinine (umol/l)* 0.8136) Where F = 1 if male, and 0.85 if female.

2 It is important to note that the ADS guidelines utilise eGFR, not CrCl, when providing advice about prescribing for people with renal impairment. While the calculation of these two measures differs, the same ‘cut offs’ have been utilised to determine if the prescriptions were consistent with guidelines. For example, the guideline recommendations for eGFR 45-60 ml/min/1.73m^2^ were applied to those with CrCl 45-60 ml/min.

3 Overall prescriptions were explored. For example, if a patient was on an appropriate dose of metformin but a dose of DPP4 inhibitor not consistent with guidelines, they were still classified as prescribed a dose not consistent with guidelines when stratified by biguanide.
