# Supplementary material for: Prescribing of diabetes medications to people with type 2 diabetes and chronic kidney disease: a national cross-sectional study
Source: BMC Fam Pract. 2019 Feb 18;20:29. doi: 10.1186/s12875-019-0915-x (PMC6378720; doi:10.1186/s12875-019-0915-x)
Supplement: Supplementary file 1 — Table S1. List of non-insulin diabetes medications available in Australia. This table summarises non-insulin diabetes medications (generic and brand names) available in Australia at the time this study was conducted. (DOCX 16 kb) [file 12875_2019_915_MOESM1_ESM.docx]

| **Generic medication** | **Brands available^[[1]](#footnote-1)^** |
| --- | --- |
| **Biguanides** |  |
| Metformin | Formet, Glucobete, Diabex, Diaformin |
| Metformin XR | Diabex XR, Diaformin XR, Metex XR |
| **Sulphonylureas** |  |
| Gliclazide | Glyade |
| Gliclazide MR | Glyade MR, Diamicron MR |
| Glibenclamide | Daonil, Glimel |
| Glimepiride | Amaryl, Aylide, Diapride, Dimirel |
| Glipizide | Melizide, Minidiab |
| **Acarbose** |  |
| Acarbose | Glucobay, Glybosay |
| **TZDs** |  |
| Pioglitazone | Acpio, Actaze, Actos, Pizaccord, Prioten, Vexazone, Pioglitazone |
| Rosiglitazone | N/A |
| **DPP4i** |  |
| Sitagliptin | Januvia |
| Vildagliptin | Galvus |
| Saxagliptin | Onglyza |
| Linagliptin | Trajenta |
| Alogliptin | Nesina |
| **Incretin mimetics** |  |
| Exenatide | Byetta |
| Exenatide XR | Bydureon |
| Liraglutide | Victoza |
| **SGLT2 inhibitors** |  |
| Dapagliflozin | Forxiga |
| Canagliflozin | Invokana (was available during the study period, but now withdrawn from market) |
| Empagliflozin^[[2]](#footnote-2)^ | Jardiance |
| **Combination formulations** |  |
| Metformin-glibenlamide | Glucovance |
| Rosiglitazone-metformin | Avandamet |
| Sitagliptin-metformin | Janumet |
| Sitagliptin-metformin XR | Janumet XR |
| Vildagliptin-metformin | Galvumet |
| Saxagliptin-metformin XR | Kombiglyze XR |
| Linagliptin-metformin | Tranjentamet |
| Alogliptin-metformin | Nesina Met |
| Dapagliflozin-metformin XR | Xigduo XR |
| Empagliflozin-metformin | Jardiamet |

**Additional file 1: Table S1**

Ref: Australian Medicines Handbook. Australian Medicines Handbook Adelaide: Australian Medicines

Handbook Pty Ltd; 2018 (online) Available from: <http://amhonline.amh.net.au/>.

1. Note – other generic brands may be available that are not listed [↑](#footnote-ref-1)
2. Empagliflozin not included in ADS guidelines. Dosing information taken from manufacturer’s Product Information.

   *Average eGFR calculated from the two most recent eGFR results within 3.5 year period prior to prescription date [↑](#footnote-ref-2)
