# Supplementary material for: Prescribing of diabetes medications to people with type 2 diabetes and chronic kidney disease: a national cross-sectional study
Source: BMC Fam Pract. 2019 Feb 18;20:29. doi: 10.1186/s12875-019-0915-x (PMC6378720; doi:10.1186/s12875-019-0915-x)
Supplement: Supplementary file 2 — Table S2. Prescription of non-insulin diabetes medications by eGFR for patients with average eGFR <60 ml/min/1.73m2. This table summarises the prescription of non-insulin diabetes medications by average eGFR consistent with stage 3a, 3b, 4 and 5 chronic kidney disease. (DOCX 24 kb) [file 12875_2019_915_MOESM2_ESM.docx]

**Additional file 2: Table S2**

| **Average eGFR (ml/min/1.73m^2^)*** | | | | | | | | | | | | | | | | | | |
| --- | --- | --- | --- | --- | --- | --- | --- | --- | --- | --- | --- | --- | --- | --- | --- | --- | --- | --- |
| **N=3505** | **45-59, n=1909** | | | | **30-44, n=1261** | | | | **15-29, n=290** | | | | | **<15, n=45** | | | | |
| **Drug/Class** | **Number of patients prescribed medication (n)** | **Recommended dose/day** | **Dose not consistent with guidelines n (%)** | **Missing dose n (%)** | **Number of patients prescribed medication (n)** | **Recommended dose/day** | **Dose not consistent with guidelines n (%)** | **Missing dose n (%)** | | **Number of patients prescribed medication (n)** | **Recommended dose/day** | **Dose not consistent with guidelines n (%)** | **Missing dose n (%)** | | **Number of patients prescribed medication (n)** | **Recommended dose/day** | **Dose not consistent with guidelines n (%)** | **Missing dose n (%)** |
| Biguanides |  |  |  |  |  |  |  |  | |  |  |  |  | |  |  |  |  |
| **Metformin** | 1664 | ≤1500mg | 691 (43.2) | 65 (3.9) | 1003 | ≤850mg | 758 (78.2) | 34 (3.4) | | 128 | Avoid | 128 (100.0) | - | | 7 | Avoid | 7 (100.0) | - |
| **Metformin XR^[[1]](#footnote-1)^** | 30 | ≤1500mg | 11 (36.7) | - | 19 | ≤1000mg | <5 | - | | <5 | Avoid | <5 | - | | - | Avoid | - | - |
| Sulphonylureas |  |  |  |  |  |  |  |  | |  |  |  |  | |  |  |  |  |
| **Gliclazide** | 528 | Use at low doses; titrate^[[2]](#footnote-2)^ | <5 | 22 (4.2) | 461 | Use at low doses; titrate^[[3]](#footnote-3)^ | <5 | 26 (5.6) | | 141 | Use at low doses; titrate | <5 | 7 (5.0) | | 25 | Avoid | 25 (100.0) | - |
| **Gliclazide MR** | 178 | Use at low doses; titrate^2^ | <5 | <5 | 175 | Use at low doses; titrate^2^ | <5 | 6 (3.4) | | 34 | Avoid | 34 (100.0) | - | | 11 | Avoid | 11 (100.0) | - |
| **Glibenclamide** | 41 | Avoid | 41 (100.0) | - | 24 | Avoid | 24 (100.0) | - | | 6 | Avoid | 6 (100.0) | - | | - | Avoid | - | - |
| **Glimepiride** | 81 | 1mg | 62 (82.7) | 6 (7.4) | 57 | 1mg | 39 (75.0) | 5 (8.8) | | 16 | Avoid | 16 (100.0) | **-** | | - | Avoid | - | - |
| **Glipizide** | 11 | 5-10mg | 6 (60.0) | <5 | 20 | 5-10mg | <5 | <5 | | 8 | 2.5mg | 7 (100.0) | <5 | | <5 | Avoid | <5 | - |
| Acarbose |  |  |  |  |  |  |  |  | |  |  |  |  | |  |  |  |  |
| **Acarbose** | 8 | 50-300mg | <5 | - | 12 | 50-300mg | - | - | | <5 | Avoid | <5 | - | | - | Avoid | - | - |
| TZDs |  |  |  |  |  |  |  |  | |  |  |  |  | |  |  |  |  |
| **Pioglitazone** | 48 | 15-45mg | - | <5 | 37 | 15-45mg | - | - | | 10 | 15-45mg | - | - | | <5 | Not recommended | <5 | - |
| **Rosiglitazone** | 7 | 2-8mg | - | - | <5 | 2-8mg | - | - | | <5 | Not recommended | <5 | - | | - | Not recommended | - | - |
| DPP4i |  |  |  |  |  |  |  |  | |  |  |  |  | |  |  |  |  |
| **Sitagliptin** | 373 | 50mg | 274 (75.9) | 12 (3.2) | 236 | 50mg | 126 (56.0) | 11 (4.7) | | 66 | 25mg | 48 (72.7) | - | | 6 | 25mg | <5 | - |
| **Vildagliptin** | 54 | 50mg | 45 (88.2) | <5 | 37 | 50mg | 29 (78.4) | - | | 6 | 50mg | <5 | - | | <5 | 50mg | <5 | - |
| **Saxagliptin** | 58 | 2.5mg | 50 (89.3) | <5 | 21 | 2.5mg | 15 (71.4) | - | | 8 | 2.5mg | 8 (100.0) | - | | - | Avoid | - | **-** |
| **Linagliptin** | 160 | 5mg | - | 8 (5.0) | 221 | 5mg | - | 12 (5.4) | | 86 | 5mg | <5 | <5 | | 20 | 5mg | - | - |
| **Alogliptin** | 10 | 12.5mg | 5 (55.6) | <5 | 5 | 12.5mg | <5 | - | | - | 6.25mg | - | - | | <5 | 6.25mg | - | - |
| Incretin mimetics |  |  |  |  |  |  |  |  | |  |  |  |  | |  |  |  |  |
| **Exenatide** | 85 | 10μgbd | <5 | 38 (45.2) | 54 | 10μgbd | <5 | 19 (38.0) | | 6 | Avoid | 6 (100.0) | - | | - | Avoid | - | - |
| **Exenatide XR** | - | 2mg/week | - | - | - | 2mg/week | - | - | | - | Avoid | - | - | | - | Avoid | - | - |
| **Liraglutide** | <5 | 0.6-1.8mg^[[4]](#footnote-4)^ | <5 | <5 | <5 | 0.6-1.8mg^[[5]](#footnote-5)^ | <5 | <5 | | - | Not recommended | - | - | | - | Not recommended | - | - |
| SGLT2 inhibitors |  |  |  |  |  |  |  |  | |  |  |  |  | |  |  |  |  |
| **Dapagliflozin** | 124 | Avoid | 124 (100.0) | - | 53 | Avoid | 44 (100.0) | - | | <5 | Avoid | <5 | - | | <5 | Avoid | <5 | - |
| **Canagliflozin** | <5 | 100mg | <5 | - | <5 | Avoid | - | - | | <5 | Avoid | <5 | - | | - | Avoid | - | - |
| **Empagliflozin^[[6]](#footnote-6)^** | 26 | 10-25mg | - | - | 8 | Avoid | 7 (100.0) | - | | - | Avoid | - | - | | - | Avoid | - | - |

1. Extended release medication not differentiated from immediate release medication in ADS table. Dosing information taken from Australian Medicines Handbook (Available from http://amhonline.amh.net.au/) [↑](#footnote-ref-1)
2. ADS guidelines and Australian Medicines Handbook do not provide a maximum recommended dose. They advise that dose reduction may be required; up to maximum dose is accepted in this study.

   Note: cell counts less than 5 suppressed due to potential confidentiality issues [↑](#footnote-ref-2)
3. ADS guidelines and Australian Medicines Handbook do not provide a maximum recommended dose. They advise that dose reduction may be required; up to maximum dose is accepted in this study.

   Note: cell counts less than 5 suppressed due to potential confidentiality issues [↑](#footnote-ref-3)
4. ADS guidelines suggest a maximum dose of 1.2mg for liraglutide but the product information advises that up to 1.8mg can be used. [↑](#footnote-ref-4)
5. ADS guidelines suggest a maximum dose of 1.2mg for liraglutide but the product information advises that up to 1.8mg can be used. [↑](#footnote-ref-5)
6. Empagliflozin not included in ADS guidelines. Dosing information taken from manufacturer’s Product Information.

   *Average eGFR calculated from the two most recent eGFR results within 3.5 year period prior to prescription date [↑](#footnote-ref-6)
